# Barriers to viral load suppression among adolescents living with HIV on anti-retroviral therapy: a retrospective study in Tanga, Tanzania

**DOI:** 10.1186/s12981-024-00622-7

**Published:** 2024-05-22

**Authors:** Stella Emmanuel Mushy, Expeditho Mtisi, Simon Mkawe, Eric Mboggo, John Ndega, Khadija I. Yahya-Malima, Denice Kamugunya, Edwin Samuel Kilimba, Boniface S. Mlay, Aisa Muya, Frida Ngalesoni

**Affiliations:** 1 Muhimbili University of Health and Allied Sciences, Dar es Salaam, Tanzania; 2 Dar Es Salaam Institute of Technology, Dar es Salaam, Tanzania; 3 Amref Health Africa, Ali Hassan Mwinyi Road, Dar es Salaam, Tanzania; 4 Center of Disease Control and Prevention, Dar es Salaam, Tanzania; 5 National Aids, Sexually Transmitted Diseases and Hepatitis Control Progralmme, Ministry of Health, Dodoma, Tanzania

**Keywords:** Adolescents, Antiretroviral therapy, Determinants, Dolutegravir, Viral load suppression, Tanzania

## Abstract

**Background:**

Despite the decreased incidence of the human immunodeficiency virus (HIV) in Tanzania, the number of adolescents living with HIV is increasing. This study aimed to describe factors independently associated with viral load non-suppression among adolescents living with HIV (ALHIV) on ART in the Tanga region.

**Methods:**

We conducted a retrospective study of routinely collected data from ALHIV on ART from October 2018 to April 2022. We extracted data from the Care and Treatment Clinics form number 2 (CTC2) database that included age, sex, BMI, World Health Organization HIV clinical disease stage, marital status, ART duration, viral load suppression, facility level, and Dolutegravir (DTG)-based regimen. We did descriptive analysis using frequencies to describe the study participants’ socio-demographic and clinical characteristics. The Cox proportional hazard regression model was used to identify factors associated with viral load non-suppression (VLS). Viral load non-suppression was defined as viral load ≥ 1000 copies/ml. A total of 4735 ALHIV on ART were extracted from CTC2, then 2485 were excluded (2186 missed viral load results, 246 were lost to follow-up, and 53 deaths).

**Results:**

2250 ALHIV on ART were tested for viral load, of whom 2216 (98.62%) adolescents were on first-line ART, and 2024 (89.96%) participants were virally suppressed, while 226 (10.04%) were virally non-suppressed. In addition, 2131 (94.71%) of participants were using a DTG-based regimen; of them, 1969 (92.40%) were virally suppressed. Not using a DTG-based regimen (HR: 9.36, 95% CI 3.41–15.31) and dispensary facility level (HR: 3.61, 95% CI 1.44–7.03) were independently associated with increased hazard for viral load non-suppression. In addition, adolescents aged between 15 and 19 years are less likely to be virally suppressed (HR: 0.55, 95% CI 0.30–0.99).

**Conclusions:**

The dispensary facility level and not using a DTG-based regimen were significantly associated with viral load non-suppression. HIV intervention strategies should ensure a DTG-based regimen utilization in all adolescents living with HIV, and techniques used by higher-level health facilities should be disseminated to lower-level facilities.

## Background

Globally, as of 2021, the number of people living with human immunodeficiency virus (HIV) was estimated at 38.4 million, with 1.7 million being children aged 0 to 14 years [1]. Within the Sustainable Development Goals (SDGs), Goal 3 focuses on ensuring healthy lives and promoting well-being for all at every stage of life, including targeting an end to the AIDS epidemic by 2030. This goal encompasses reducing new HIV infections, ensuring universal access to prevention, treatment, care, and support services for HIV, and achieving viral suppression for individuals living with the virus [2]. In 2021, 85% of people living with HIV globally knew their status, 75% were accessing treatment, and 68% had achieved a low viral load [1]. However, these achievements vary significantly across different population groups, with adolescents in sub-Saharan Africa (SSA) notably falling behind in viral load suppression (VLS) [1]. The World Health Organization (WHO) defines adolescents as individuals aged 10 to 19 years [3].

In SSA, three in every four new HIV infections are young people, and six in every seven new infections among adolescents aged 15–19 years were among women, accounting for 63% of all new HIV infections [1]. Adolescents in SSA continue to be disproportionately affected, contributing the most significant part of the overall HIV suppression failure prevalence of 47% [4]. Information, especially regarding the prevalence of VLS among adolescents (10–19 years) living with HIV on ART, remains scarce in Tanzania.

Viral load suppression is a crucial marker of therapy efficacy in PLHIV. To be termed that the virus is suppressed, the VLS is thought to be < 1000 copies/ml of plasma and unsuppressed when ≥ 1000 copies/ml of plasma [5]. The fundamental goal of HIV infection treatment is to suppress HIV, which will ultimately increase survival, improve quality of life, and reduce HIV transmission [5]. In addition, follow-up of the VLS status among adolescents is crucial for early identification of treatment failure for patients needing intensive adherence counselling and prevents the occurrence of drug resistance [6]. Therefore, UNAIDS launched the 95–95–95 targets in 2014, the “Third 95” target aiming to increase viral load suppression to 95% of all HIV-infected individuals on ART by 2030 [4]. The “Third 95” target focused on defeating HIV/AIDS because HIV patients with VLS have low chances of transmitting infections to others [4].

Several efforts have been undertaken to address the issue of viral non-suppression among people living with HIV (PLHIV) in Tanzania. These efforts encompass enhanced treatment adherence programs offering education, counseling, and support services, alongside increased availability and accessibility of viral load (VL) testing services to monitor antiretroviral therapy (ART) effectiveness and detect non-suppression early. Healthcare providers have been provided with training on the importance of VL monitoring, result interpretation, and appropriate actions in case of non-suppression. Additionally, newer, more potent ART medications like DTGs are being promoted, while communities affected by HIV/AIDS are being engaged to raise awareness about the importance of ART adherence. Advocacy for policies supporting comprehensive HIV/AIDS care and treatment, including strategies to improve viral load suppression rates, is also ongoing [7–10].

In Tanzania, among people living with HIV (PLHIV) aged 15 years and older who are receiving antiretroviral therapy (ART), only 37.9% achieved viral load suppression (VLS) among adolescents and young adults aged 15–24 years [9]. Despite the concerning treatment outcomes regarding VLS among adolescents receiving ART in Tanzania, their circumstances are often overlooked. This oversight may stem from how age is categorized in Tanzania, where adolescents between 10 and 14 years are grouped with children aged 0–14, while those between 15 and 19 years are considered adults [9, 10]. This classification system limits the availability of specific HIV treatment outcome data for adolescents aged 10–19 years. Furthermore, while adolescents constitute a significant proportion of individuals with virally non-suppressed HIV in Tanzania, there is limited published data on treatment outcomes for adolescents aged 10 -19 years regarding VLS and associated factors.

Adolescents are in the transition phase from childhood to adulthood, so the physiological changes resulting from puberty put them at different risks, including behavioural changes that might interrupt ART adherence and delay achieving the “Third 95” target. Lack of special attention to this population segment puts them at high risk of HIV-related morbidity and mortality. Understanding HIV treatment outcomes for adolescents (10–19 years) and associated factors is critical to urgently identify and inform program interventions to optimize ART outcomes to help achieve the “Third 95” target of suppressing viral load to 95%.

## Methods

### Study design and setting

We conducted a retrospective review of routinely collected data from ALHIV on ART from October 2018 to April 2022 from the Tanga region, Tanzania. The current HIV prevalence in Tanga is 5% (3.7 men: 6.2 females). Administratively, Tanga has eight districts (Tanga, Muheza, Korogwe, Lushoto, Handeni, Pangani, and Kilindi). The study sites were all HIV/AIDS care and treatment districts' health care services facilities in Tanga supported by Amref Health Africa Tanzania. Tanzania's healthcare system blends public and private providers, with significant variations in access and quality of care across different regions of the country. The Ministry of Health, oversees policy, regulation, and service coordination. Primary care, offered by dispensaries, health centers, and district hospitals, delivers basic preventive and curative services. Regional and national referral hospitals provide specialized care. CTC services are integrated into the primary healthcare system, which includes dispensaries, health centers, and district hospitals. This integration ensures that HIV/AIDS care is accessible at the community level and provides a continuum of care for individuals living with HIV/AIDS [11].

### Study population and sampling

**Fig. 1:**
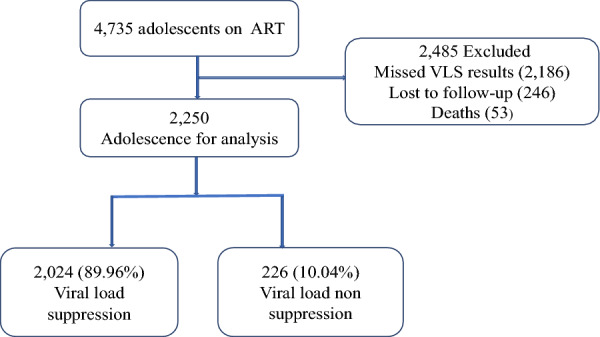
Flow chart of CTC2 data for adolescence from 1st October 2018 to April 2022 in Tanga region, Tanzania

We included adolescents aged 10–19 years on antiretroviral therapy (ART) in the selected care and treatment districts’ health services facilities in the Tanga region supported by Amref Tanzania and on ART for at least six months. We included ALHIV on ART for at least six months because the first VL test is done in the sixth month after initiating ART. From the CTC2 database, 4735 ALHIV were on ART, then excluded 2485 adolescents (2186 missed viral load results, 246 lost to follow-up, and 53 deaths). After data cleaning, 2250 adolescents were included in the final analysis (Fig. 1). The viral load suppression reported for ALHIV relied on the participants on ART for at least six months, who were followed up over a particular time until they had non-viral load suppression or a competing event (attrition and death).

### Study variables and analysis

After a thorough review of similar studies that assessed barriers to viral load suppression (VLS) among ALHIV on ART, we considered the following variables as possible determinants of VLS; age (10–14; 15–19); sex (female, male); body mass index (BMI) (< 18.5, 18.5–< 25, 25–< 30, 30 +); WHO HIV clinical disease stage (I, II, III, IV); marital status (cohabiting, divorced, married, single and widow); ART duration (< = 1, > 1 years); ART regimen (first-line, second-line). First, we did a descriptive analysis to provide basic information about the variables of interest in our study to highlight potential relationships between the variables. Numbers and frequencies were used to describe categorical variables, while means/medians and interquartile ranges were used to describe continuous variables. Next, we did a bivariate analysis to ascertain the degree of association between each independent and dependent variable.

We used Cox proportional hazard regression model to describe the hazard ratio of participants with viral load non-suppression from the date of ART initiation. We used Kaplan–Meier curves to estimate the cumulative incidence of viral load non-suppression. The death date and attrition marked the end time of viral load non-suppression. We included the variables in bivariate analysis with a p ≤ 0.2 in the Cox hazard regression model as potential confounders on a two-sided test with a significant p-value less than or equal to 0.05. We used the missing indicator method to handle missing data. We used the SAS statistical software package version 9.3 to perform data analysis (Cary, NC, USA).

## Results

### Study sample characteristics

A total of 2250 ALHIV on ART for at least six months, with results of the last viral load, were included for analysis. The median age of the participants was 15.2 years (Interquartile Range [IQR] = 12.8–17.2). Table 1 shows that half of the participants (51.56%) were aged 15–19 years and females (51.33%). More than half (58.58%) of the participants attended the health centre facility level. Results also showed that most participants were on a first-line drug regimen (98.62%), and the majority (95.29%) were on ART for over one year. A great proportion of participants (89.96%) were virally suppressed, and 94.71% of ALHIV on ART were using a DTG-based regimen; of the DTG-based regimen users, 92.40% were virally suppressed. Most participants reported being single (75.34%), and 76.67% had a BMI of 18.5 – < 25 kg/m^2^. Nearly two-thirds of the participants were in WHO clinical stage III (60.35%).

**Table 1:** Socio-demographic and clinical characteristics and their association with viral non-load suppression

Study sample characteristics	Viral load non-suppression		Total	p-value
	No (%)	Yes (%)		
Median age (IQR)	15.2 (12.8–17.2)			
Age group, years				
10–14	986 (90.46)	104 (9.54)	1090 (48.44)	0.4415
15–19	1038 (89.48)	122 (10.52)	1160 (51.56))	
Sex				
Female	1056 (91.43)	99 (8.57)	1155 (51.33)	0.0170*
Male	968 (88.40))	127 (11.60)	1095 (48.67)	
Marital status				
Cohabiting	413 (89.01)	51 (10.99)	464 (22.52)	0.4558
Married	32 (86.49)	5 (13.51)	37 (1.80)	
Single	1413 (91.04)	139 (8.96)	1552 (75.34)	
Widow	6 (85.71)	1 (14.29)	7 (0.34)	
Facility level				
Dispensary	317 (89.55)	37 (10.45)	354 (20.74)	< 0.0008***
Health center	913 (91.30)	87 (8.70)	1000 (58.58)	
Hospital	297 (84.14)	56 (15.86)	353 (20.68)	
BMI, kg/m^2^				
< 18.5	83 (87.37)	12 (12.63)	95 (4.22)	
18.5 – < 24	1550 (89.86)	175 (10.14)	1725 (76.67)	0.7549
25– < 29	156 (90.70)	16 (9.30)	172 (7.64)	
30 +	235 (91.09)	23 (8.91)	258 (11.47)	
WHO clinical stage				
I				
II	241 (90.94)	24 (9.06)	265 (13.47)	0.8557
III	250 (91.58)	23 (8.42)	273 (13.88)	
IV	1068 (89.97)	119 (10.03)	1187 (60.35)	
ART duration (yrs.)				
< = 1	92 (86.79)	14 (13.21)	106 (4.71)	
> 1	1932 (90.11)	212 (9.89)	2144 (95.29)	0.2671
Regimen				
First-line	2003 (90.39)	213 (9.61)	2216 (98.62)	< 0.0001***
Second-line	21 (67.74)	10 (32.26)	31 (1.38)	
DTG-based regimen				
No	55 (46.22)	64 (53.78)	119 (5.29)	< 0.0001***
Yes	1969 (92.40)	162 (7.60)	2131 (94.71)	
Viral load status	2024 (89.96)	226 (10.04)	2250 (100)	

^*^p-value is less than 0.05

^***^p-value is less than 0.001

## Socio-demographic and clinical characteristics of the participants with viral load non-suppression

As illustrated in Table 1, more males (11.60%) compared to females (8.57%) were virally non-suppressed. The age group 15–19 years accounted for 10.52% viral load non-suppression compared to the age group 10–14 years (9.54%). Furthermore, adolescents on the first-line regimen accounted for 9.61% viral load non-suppression compared to 32.26% on the second-line regimen. More virally non-suppressed participants (15.86%) were observed from the dispensary than at the health center and hospital facility levels.

**Fig. 2:**
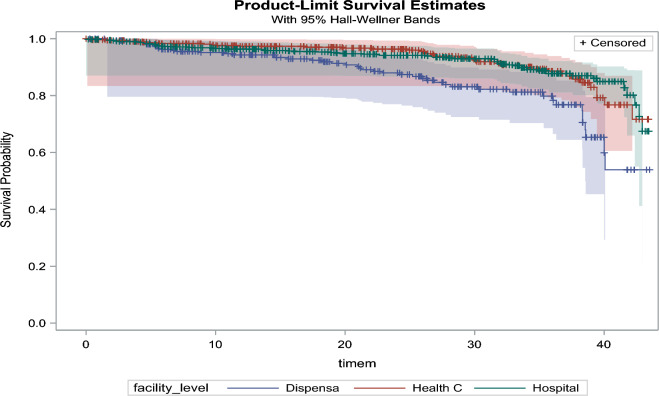
Survival probability among adolescents by facility level

**Fig. 3:**
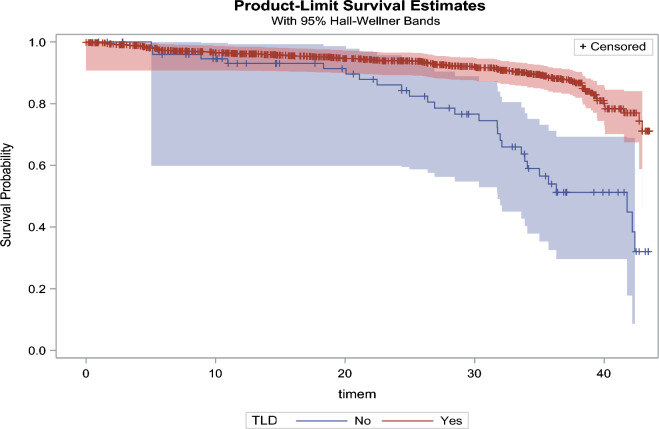
Survival probability among adolescents by DTG-based regimen

Covariates-adjusted Kaplan Meir plots for the overall viral load non-suppression by the facility and DTG-based regimen suppression categories are presented in Figs. 2 and 3

### Factors independently associated with viral load non-suppression

Table 2 shows that the likelihood of being virally non-suppressed for participants not on a DTG-based regimen was more than nine times at increased hazard (HR: 9.36, 95% CI 3.41–15.31) than those on a DTG-based regimen, p < 0.0001. Additionally, participants in care and treatment at the dispensary level (HR: 3.61, 95% CI 1.44–7.03) had 3.6 times increased hazard of being virally non-suppressed than participants in the health center and the hospital, p = 0.007. Furthermore, the age of 15–19 years (HR:0.55, 95% CI 0.30–0.99) is less likely to be virally non-suppressed than the age of 10–14 years, p = 0.04376.

**Table 2:** Factors independently associated with viral load non-suppression among ART-experienced PLHIV adolescents

Characteristics	Unadjusted HR 95% CI	p-value	Adjusted HR 95% CI	p-value
Age group (yrs.)				
10–14	Reference	1	Reference	0.0474*
15–19	0.90 (0.88–1.03)	0.1920*	0.55 (0.30–0.99)	
Sex				
Female	Reference	1	Reference	0.4376
Male	1.40 (1.06–1.85)	0.0174^*^	1.26 (0.70–2.28)	
Marital status				
Cohabiting	1.25 (0.89–1.76)	0.7564		
Married	Reference	1		
Separated	1.59 (0.61–3.52)			
Widow	1.69 (0.20–9.56)			
BMI group, kg/m^2^				
< 18.5				
18.5–24	1.28 (0.69–2.39)	1		
25–29	Reference	0.4540		
30 +	0.91 (0.53–1.55)			
WHO clinical stage				
I				
II	Reference	1		
III	0.92 (0.51–1.68)	0.5800		
IV	1.12 (0.71–1.77)			
ART duration (yrs.)				
< = 1	1.39 (0.78–2.48)	0.2681		
> 1	Reference	1		
Regimen				
First-line	Reference	1	Reference	
Second-line	4.48 (2.08–8.63)	< 0.001^***^	2.17 (0.22–3.74)	0.7685
DTG-based regimen				
Yes	Reference	1	Reference	1
No	6.14 (4.62–10.13)	< 0.001^***^	9.36 (3.41–15.31)	< 0.001^***^
Facility level				
Hospital	Reference	1 0.003^**^	Reference	
Health centre	0.89 (0.59–1.34)		0.78 (0.40–1.54)	0.0521
Dispensary	1.84 (1.24–2.74)		3.61 (1.44–7.03)	0.007^***^

^*^ p-value is less than 0.05

^***^ p-value is less than 0.001

## Discussion

This study investigated the factors associated with viral load non-suppression among adolescents living with HIV (ALHIV) on ART in care and treatment health services facilities in Tanga. Anti-retroviral therapy (ART) aims to suppress viral replication to reduce the patient’s viral load (VL) to undetectable levels [5]. This reduction of viral load prevents further damage to the body’s immune system and restores and maintains the quality of life [5]. Therefore, early enrolment into care, retention, and good adherence to ART are vital components of AIDS and HIV transmission prevention.

The current study found that 226 participants (10.04%) were virally non-suppressed. Factors independently associated with viral load non-suppression included not using a DTG-based regimen and receiving services at the dispensary level. Additionally, adolescents aged 15–19 years were less likely to achieve viral suppression.

The viral load non-suppression prevalence among adolescents in this study was lower than the national prevalence of 17.7% reported in 2016 [69. This rate is lower than the studies in Uganda, Swaziland, and South Africa, at 31.4%, 16%, and 15%, respectively [12–14]. However, this observed viral load non-suppression rate falls short of the UNAIDS’ 95-95-95 targets. The observed lower viral load non-suppression rate in the current study may be related to the rollout of a DTG-based regimen for all PLHIV that could have helped to influence viral load suppression. The present study showed that ALHIV on a DTG-based regimen was significantly virally suppressed. In addition, PLHIV on ART, once detected to have a high viral load, receive three consecutive sessions of enhanced adherence counselling that might have contributed to the improved adherence and eventually reduced viral load suppression. Our findings suggest that the WHO target of 95% viral suppression by 2025 is possible for ALHIV in Tanzania if HIV services are improved.

The DTG-based regimen is more effective in suppressing viral load than the non-DTG-based regimen [15–17]. However, in the present study, most virally non-suppressed did not use a DTG-based regimen. In Tanzania, the use of ART with a DTG-based regimen was rolled out in 2019. Unfortunately, to the best of our review, limited published studies looked at the association between viral load suppression and the use of a DGT-based regimen.

The ALHIV accessing care and treatment at the dispensary facility level had a higher risk of being virally non-suppressed than those who attended HIV care and treatment at the hospital and health center facilities. The possible reason for this observation could be limited strategies to follow up with patients at the lower health facility levels than higher facilities. However, limited studies examined the association between facility-level attendance and viral load suppression.

Evidence points to a connection between sex and the suppression of viral load. For instance, one study indicated that males were more likely than females to be virally suppressed [18]. In contrast, another study found that females were more likely than males to be virally suppressed [19]. However, our study found that males were less likely to be virally suppressed than females. Implying that implemented efforts to improve VLS should put more efforts on reaching men. The outcome may be related to their reported lower treatment adherence rates. It is difficult to link and keep males in ART therapy [20] and have them tested for HIV since they also exhibit poor treatment-seeking behavior [19]. This finding has been evidenced in the first studies that analysed ART treatment after five years of ART initiation in Tanzania. Excess mortality in men was rooted in poor health-seeking behavior and non-compliance with medication [21]. Males are also discouraged from obtaining healthcare services due to a shortage of male-friendly services [20, 22, 23].

### The study limitations and recommendations

The longitudinal nature of this study gave room to calculate the rate and barriers that independently hindered viral load suppression among ALHIV in Tanga. However, this study used secondary data that limited information on important variables like pregnant status, ART adherence, support from significant others, and disclosure status, which are reported to delay viral load suppression. Using secondary data presented challenges in the quality, completeness, and missing data that we mitigated by employing statistical and data management techniques to ensure data quality. In addition, the VLS success observed in this study was only for those whose viral load testing was possible, which might have underrepresented adolescents in the Tanga region.

## Conclusions

Viral load non‐suppression among ALHIV was almost lower than the national average. Not using a DTG-based regimen, attending care and treatment at the dispensary facility level, and age group of 15–19 years were independently associated with viral load-non-suppression among ALHIV. Therefore, adolescent-friendly strategies are essential, especially at the dispensary facility level, to help improve viral load suppression to foster the attainment of the UNAIDS's third 95 target. In addition, program implementers should improve the accessibility of a DTG-based regimen to all PLHIV on ART in the country. Furthermore, we recommend innovative interventions to ensure ART adherence and retention that would ultimately improve VLS.

## Data Availability

The datasets used and/or analyzed during the current study are available from the corresponding author upon reasonable request.

## Abbreviations

AIDS: Acquired immunodeficiency syndrome
ALPLHIH: All people living with HIV
ART: Antiretroviral therapy
CTC2: Care and treatment clinics form number two
DTG: Dolutegravir
HIV: Human immunodeficiency virus
PLHIV: People living with HIV
SSA: Sub-Saharan Africa
WHO: World Health Organization
